# Editorial: The use of extended realities providing better patient outcomes in healthcare

**DOI:** 10.3389/fmed.2024.1380046

**Published:** 2024-02-19

**Authors:** Donovan Jones, Shanna Fealy, Darrell Evans, Roberto Galvez

**Affiliations:** ^1^School of Medicine and Public Health, The University of Newcastle, Callaghan, NSW, Australia; ^2^Faculty of Science, Charles Sturt University, Wagga Wagga, NSW, Australia; ^3^Department of Psychology, Carle Illinois College of Medicine, University of Illinois at Urbana-Champaign, Champaign, IL, United States

**Keywords:** extended realities, virtual reality, medical technology (Med-Tech) innovations, augmented (virtual) reality, transformative technologies

The integration of cutting-edge technologies such as virtual reality (VR), augmented reality (AR), and mixed reality (MR), often collectively referred to as Extended Reality (XR), is becoming widely accepted as a promising innovative approach to primary health care (1). Recent developments in XR and patient-centered co-design have ushered in a new era of possibilities, challenging traditional approaches to healthcare management. In this editorial, we explore innovative XR studies published in this Research Topic: a novel MR system to manage phantom pain in-home (Annapureddy et al.); the utilization of XR with reinforcement learning in addressing social anxiety for patients with Fragile X Syndrome (Stasolla et al.); exploration of patient-centered design for patients with post-stroke depression (Li et al.); and work on digital health promotion using AR with motion detection for the wellbeing of elderly patients (Sit et al.).

Annapureddy et al. introduce a pilot clinical trial focused on a novel MR system designed to manage phantom pain in the comfort of the patient's home. Phantom pain, a complex and often debilitating condition experienced by amputees, has long been a challenging aspect of post-amputation recovery. MR for in-home phantom limb pain management created a virtual environment that mirrors the intact limb onto the amputated limb within the MR systems view (Annapureddy et al.). This virtual environment allows the user to engage in interactive games that afford different lower limb movement. The results from this pilot demonstrated an innovative MR solution to phantom limb pain, providing pain relief and a possible reduction in pharmacological use (Annapureddy et al.). Additionally, this study shows the potential of MR use beyond amputees, offering hope for those suffering from other chronic pain conditions. As healthcare continues to navigate an opioid crisis, non-pharmacological interventions such as MR can provide a crucial innovative alternative, shaping the future of pain management.

Moving beyond pain management, Stasolla et al. used a combination of XR and reinforcement learning in patients suffering from Fragile X Syndrome, and demonstrated a significant stride toward the versatility of extended reality in addressing complex neurological disorders. Fragile X Syndrome is a rare genetic disorder associated with social anxiety and poses unique challenges in both assessment and rehabilitation. The innovative assessment tool and rehabilitative strategy developed through the fusion of XR and reinforcement learning provide a patient-centered tailored approach. Stasolla et al. found that this fostered a more effective and personalized treatment, which is capable of learning from the specific performance of patients through algorithms and parameters that are continuously adapted to the learning experience. This study highlights the importance of interdisciplinary collaboration in XR while pushing the boundaries of what technology can achieve in the delivery of mental health modalities, opening the possibilities for XR use with other mental health conditions.

The research by Li et al. on patient-centered design for patients with post-stroke depression employs MR with structural equation modeling (SEM) and comprehensive evaluation. Structural equation modeling can estimate the relationship between multiple potential variables, retain and remove measurable variables with weak correlations, and is suitable for Virtual Reality Rehabilitation Landscape (VRTL) design based on Reality Based Interaction (RBI) theory (Li et al.). As a result, using MR with SEM, researchers may be afforded insights into the intricate relationships between various factors influencing post-stroke depression and the use XR for rehabilitation. In general terms, the construction of the comprehensive evaluation system of the VRTL based on RBI and SEM is an attempt to integrate XR technology into the traditional medical system, thereby laying a foundation for the future large-scale clinical application of XR in post-stroke depression (Li et al.).

With the acceleration of population aging, the use and application of XR present many possibilities in providing simple and effective interventions for older people. As described by Sit et al., combing gerontology with a technology known as gerontechnology has the ability to promote quality of life in older adults. The research by Sit et al. on the development and evaluation of XR digital health promotion game booths in Hong Kong draws attention to the role of AR and motion detection in promoting wellbeing in the older population. The integration of technology, specifically AR, and motion detection provides an engaging and interactive platform for health promotion, outlining the potential of gerontechnology in enhancing the quality of life for the elderly and promoting physical and mental wellbeing in a holistic manner.

Across these studies, several themes emerge; digital health technologies that personalize and tailor to individual patient needs and the development of XR using co-design to enhance efficacy, as demonstrated in post-stroke depression and Fragile X Syndrome management (Li et al.).

Technology integration, including the integration of XR in healthcare, offers innovative ways to manage complex conditions like phantom limb pain and social anxiety (Annapureddy et al.). User engagement that utilizes interactive and immersive experiences shows positive outcomes for elderly wellbeing and rehabilitation, which may also afford better engagement and positive health outcomes (Sit et al.). Overall, and most significantly, these studies demonstrate that XR technologies hold potential for broader application in various healthcare domains, from chronic pain management to mental health and geriatric care, and show that further research is necessary.

The convergence of XR and patient-centered design is reshaping the landscape of healthcare. The studies highlighted characterize the power of technology to address diverse challenges, from managing phantom limb pain and social anxiety to post-stroke depression and geriatric wellbeing. As we embrace these advancements, it is easy to see a future where such technologies can be used to assess, diagnose, and treat various conditions, opening up possibilities for more individualized care and increased access to rural and remote communities. However, as with all medical advancements, it is crucial to also ensure that ethical considerations, accessibility, and inclusivity are integral parts of the technological revolution in healthcare, as well as developing a solid evidence-based approach for such treatments and interventions. The journey toward a more technologically advanced and patient-centric healthcare system is underway, and the results are promising. The future of healthcare is not just about treating illnesses but empowering individuals to lead healthier and more fulfilling lives; technology will play an evolving role.

## Author contributions

DJ: Conceptualization, Writing—original draft, Writing—review & editing. SF: Conceptualization, Writing—original draft, Writing—review & editing. DE: Conceptualization, Writing—original draft, Writing—review & editing. RG: Conceptualization, Writing—original draft, Writing—review & editing.
